# Care Seeking Behaviour and Barriers to Accessing Services for Sexual Health Problems among Women in Rural Areas of Tamilnadu State in India

**DOI:** 10.1155/2014/292157

**Published:** 2014-03-20

**Authors:** Rejoice Puthuchira Ravi, Ravishankar Athimulam Kulasekaran

**Affiliations:** ^1^Institute of Rural Health Development, Kerala 686 535, India; ^2^Department of Population Studies, Annamalai University, Tamil Nadu 608 002, India

## Abstract

*Background*. Sexually transmitted infections (STIs) may be either asymptomatic or symptomatic. Regardless of the presence or absence of symptoms all STIs can lead to major complications if left untreated. *Objective*. To assess the care seeking behaviour and barriers to accessing services for sexual health problems among young married women in rural areas of Thiruvarur district of Tamil Nadu state in India. *Methods*. A community based cross-sectional study was conducted in 28 villages selected using multistage sampling technique for selecting 605 women in the age group of 15–24 years during July 2010–April 2011. *Results*. The prevalence rate of reproductive tract infections (RTIs) and STIs was observed to be 14.5% and 8.8%, respectively, among the study population. Itching/irritation over vulva, thick white discharge, discharge with unpleasant odor, and frequent and uncomfortable urination were most commonly experienced symptoms of sexual health problems. Around three-fourth of the women received treatment for sexual health problems. Perception of symptoms as normal, feeling shy, lack of female health workers, distance to health facility, and lack of availability of treatment were identified as major barriers for not seeking treatment for RTIs/STIs. *Conclusion*. Family tradition and poor socioeconomic conditions of the family appear to be the main reasons for not utilizing the health facility for sexual health problems. Integrated approach is strongly suggested for creating awareness to control the spread of sexual health problems among young people.

## 1. Introduction

Sexually transmitted infections (STI) are now recognized as a serious global threat to the health of populations. Sexually transmitted infections have a major negative impact on sexual and reproductive health worldwide. According to 2008 WHO estimates, 499 million new cases of curable STIs (syphilis, gonorrhoea, chlamydia, and trichomoniasis) occur annually throughout the world in adults aged 15–49 years [1]. In India, it is estimated that 5% of the adult population has STI symptoms [2]. RTIs and STIs are affecting health, fertility, infant mortality, postorbital and puerperal sepsis, ectopic pregnancy, fatal and prenatal death, cervical cancer, infertility, chronic physical pain, emotional distress, and social rejection in women. There are 340 million new cases of largely treatable sexually transmitted bacterial infections occurring annually [3], 100 million of them among young people. Many go untreated due to difficulties in diagnosis and lack of access to competent, affordable services. Many of these infections increase the risk of HIV transmission.

Reproductive tract infections (RTIs) are recognized as a major public health problem and rank second after maternal morbidity and mortality as the cause of healthy life loss among sexually active women of reproductive age in developing countries [4]. These RTIs carry a high economic burden as well as enormous health consequences. RTIs have overlapping categories called endogenous, sexually transmitted and iatrogenic, reflecting how they are acquired and spread [5]. RTIs are most important causes of maternal and perinatal morbidity and mortality. Serious complications of these RTIs include entopic pregnancy, pelvic inflammatory diseases, preterm labor, miscarriage, still birth, congenital infection, infertility, genital cancer, and risk of HIV infection [6]. Women living in medium economic level and low socioeconomic status were all related to having RTIs symptoms [7, 8]. Poverty and marginalization were associated with STIs and bacterial vaginosis [9].

Generally women with self-reported symptoms of sexual morbidity do not seek treatment due to existing taboos and inhibitions regarding sexual and reproductive health. They hesitate to discuss the reproductive problem especially due to shame and embarrassment [10]. Untreated infection can not only lead to pelvic inflammatory disease, ectopic pregnancy, infertility, and cervical cancer but also fetal loss, health problem of new born, and increased risk of HIV infection. In addition to health consequences, women experience social consequences in terms of emotional distress related to gynecological morbidity. A recent study of young married women aged 16–22 years in a rural community in Tamil Nadu reports a very high level of morbidity. The study shows that more than half of the women were suffering from at least one or more RTIs/STIs. Clinical examination also confirmed STIs among the majority of them [11]. Adolescent women in India and Nepal report relatively high rates of gynecological morbidities, especially in the settings where girls have limited access to adequate health care [12].

### 1.1. A Profile of Scheduled Castes (SC) Population in India

The Indian caste system is a highly complex institution, though social institutions resembling caste in one respect or another are not difficult to find elsewhere, but caste is an exclusively Indian phenomenon. The “scheduled castes” is the legal and constitutional name collectively given to the groups which have traditionally occupied the lowest status in Indian society and the Hindu religion which provides the religious and ideological basis for a “disadvantaged” group, which was outside the caste system and inferior to all other castes [13]. At present, the scheduled castes in India constitute around 16.8% of the total population. Almost one-third of them live below poverty line and do not have access even to the basic needs like food, clothing, and shelter; they constitute a major part of our labor force and are generally engaged in petty occupations like agriculture labor, construction work, hawking, and other low grade jobs [14]. There is a general consensus that the health status of the scheduled castes population is very poor and the worst [15]. Under this circumstance, the present study made an attempt to assess the care seeking behaviour and barriers to accessing services for sexual health problems among young married women in rural areas of Thiruvarur district of Tamil Nadu state in India.

## 2. Materials and Methods

### 2.1. Study Area

According to 2001 census, Thiruvarur district was the highest scheduled castes populated district and also backward district in Tamil Nadu state. All women were living with their husbands and had given at least one birth one year prior to the survey.

### 2.2. Study Design

A community based cross-sectional study was conducted in 28 villages selected using multistage sampling technique for selecting 605 women in the age group of 15–24 years during July 2010–April 2011.

### 2.3. Selection of the Blocks

Thiruvarur district had totally ten blocks, which comprise 573 revenue villages. In the first stage, five blocks were selected which represent the geographical distribution of the study district. The selected blocks were Nannilam from north, Thiruvarur from east, Tiruturaipundi from south, Valangaiman from west, and Mannargudi from central part of the study district.

### 2.4. Selection of the Villages

There were 352 revenue villages in these selected five blocks. In the second stage, all the villages which had 50 percent of scheduled castes population were selected. That is, 87 villages were selected. For covering the entire block, one-third of the villages (5/6 villages) were selected from each block by simple random sampling method. Thus, 28 villages were selected for the research purpose.

### 2.5. Selection of the Respondents

In the third stage, house listing operation was carried out prior to the data collection to provide the necessary frame for selecting the households for the study. Totally, 6376 houses were listed in all the five blocks. Identification of eligible young married women (15–24 years) in each household was the next step in the research. There were 1164 households with the target population (39 households had two couples). Totally, 1203 women in the age group of 15–24 were identified.

Systematic random sampling technique was applied for selecting 21/22 respondents from each village. In order to take care of nonresponse due to various reasons, an extra 10% of respondents were included in the sample. That is, 661 respondents were selected for the interview. Totally, 605 respondents completed the interview and 32 respondents declined to participate in the interview. The response rate of the research study was 91.5%.

### 2.6. Data Collection Tools

The respondents were assessed using a structured interviewer administered questionnaire which was pretested in Chidambaram Taluk near Annamalai University, about 102 km away from Thiruvarur district. The authors collected all the required data from the respondents with the help of local trained female workers.

### 2.7. Data Analysis

Data were entered and analyzed using SPSS software version 17. Categorical variables were presented as frequencies and percentages. Bivariate analysis involved the use of the Chi-square test for assessing the significance of associations between care seeking behavior of women and sociodemographic variables.

### 2.8. Ethics Review

The syndicate review board at Annamalai University, Tamilnadu state, India, has approved the research entitled “reproductive and sexual health status of scheduled castes youth in Thiruvarur district, Tamilnadu, India” for the degree of Doctor of Philosophy (PhD) in Population Studies with effect from July 2012.

### 2.9. Study Setting

See Figure 1.

## 3. Results

### 3.1. Incidence and Treatment of Reproductive Tract Infections (RTIs)

All the respondents were asked whether they had experienced any kind of symptoms of RTIs for the last six months prior to the survey and the results are tabulated in Table 1. The result reveals that 14.5% of the scheduled castes (SC) women experienced RTIs. Among women who experienced RTIs, around nine percent of women reported that they suffered “itching/irritation over vulva” and 4.6% of women experienced “thick white discharge” and “pain in lower abdomen” (not related to menses). Result shows that women who had “itching/irritation over vulva”, about three-fourth of them had undergone the treatment (75.9%). More than ninety-five percent of the women who had experienced the “thick white discharge” had taken treatment. Around eighty percent of women had sought treatment for their “pain during urination” (81.2%), and also three-fifth of women had undergone treatment for their “pain in lower abdomen” (60.7%). It was observed that the majority of the women in the study area had the tendency of seeking treatment for their RTI problems (80.7%).

### 3.2. Care Seeking Behaviour of Women Who Experienced RTIs according to Their Background Characteristics

Table 2 shows the percentage of women who sought treatment of RTIs according to their background characteristics. The result indicates that younger women were more likely to receive treatment for RTIs (86.5% among 18–20 years) than those aged 24 years (78.3%). Women's education had a positive relationship with treatment seeking behaviour. The nonagricultural laborers (84.2%) were more likely to receive treatment of RTI problems than nonworking women (66.7%). The women in households in the highest standard of living index (SLI) were more likely to receive treatment for their RTIs (100%) than women in households in the lowest SLI (71.9%). The women whose age at marriage was 22 and above were more likely to receive treatment of RTIs (80.0%) than those whose age was less than 18 years (60%). The proportion of women who sought treatment of RTIs decreased sharply by birth order. The higher birth order pregnancy women were less likely to receive treatment of RTIs (57.1%) than lower birth order pregnancies (87.2%). About 91.7% of women received treatment of RTIs who reside within one Km radius of healthcare facilities than women who reside four Km away from healthcare facilities (75.5%).

### 3.3. Incidence and Treatment of Sexually Transmitted Infections (STIs)

Table 3 reveals that 8.8% of women experienced STIs in the study area. Among women who experienced STIs, only 4.6% of women reported that they suffer “discharge with unpleasant odor” followed by “frequent and uncomfortable urination” (3.6%). Meager portion of women stated that they experienced “pain during sexual intercourse” (3.1%). A significant portion of women had sought treatment (77.4%) for their sexual health problems. About 82.1% of them had undergone the treatment for “discharge with unpleasant odor” problems. More than eighty percent of the women who had experienced the “frequent and uncomfortable urination” had taken treatment and another 73.7% of women had sought treatment for their “pain during sexual intercourse” problem. Two-third of women received treatment for their “spotting after sexual intercourse” problem (66.7%).

### 3.4. Care Seeking Behaviour of Women Who Experienced STIs according to Their Background Characteristics

Table 4 shows the percentage of women who sought treatment of STIs according to their background characteristics. It is observed that younger women were much more likely to receive treatment for their STIs than the older women. The result depicts that women in age group 18–20 were more likely to receive treatment of STIs (80.0%) than those aged 24 years (75.0%). Overwhelming proportion of women received treatment of STIs who completed secondary education (85.7%) than those who completed primary education (70.0%) and illiterates (66.7%). The treatment of STIs was more pronounced among employed women than among their counterparts. The result shows that all women who were working in nonagricultural sector sought treatment of STIs (100%), whereas this proportion among women working in the agricultural sector was 63.3% and not working women was 75.0%. The finding indicates that women in households in the highest standard of living index (SLI) were more likely to receive treatment of STIs (100%) than women in households in the lowest SLI (72.7%) (*χ* ^2^ = 17.17 and *P* = .006). The women whose age at marriage was 22 and above were less likely to receive treatment of STIs (83.3%) than those aged less than 18 years (100%). The higher birth order among women was less likely to receive treatment of STIs (66.7%) than lower birth order (83.3%). Women's exposure to mass media had a negative relationship with treatment seeking behaviour. Around 87% of women received treatment of STIs who reside within one Km radius of healthcare facilities than women who reside four Km away from healthcare facilities (75%).

## 4. Discussion

Through this study, authors tried to highlight the treatment seeking behavior of women regarding RTIs and STIs in rural areas of Tamil Nadu state. The prevalence rate of RTIs and STIs was observed to be 14.5% and 8.8%, respectively, among the population in the study area. Itching/irritation over vulva, thick white discharge, and pain in lower abdomen (not related to menses), discharge with unpleasant odor, and frequent and uncomfortable urination were most commonly experienced symptoms of sexual health problems. Unfortunately, symptoms and signs of many infections may not appear until it is too late to avoid such consequences and damage to the reproductive organs. The morbidity associated with RTIs also affects the economic productivity and quality of life of many individual women and men and, consequently, of whole communities [16].

Around three-fourth of the women received treatment for RTIs/STIs; all were treated in public health institutions. Perception of symptoms as normal, feeling shy, lack of female health workers, distance to health facility, and lack of availability of treatment were identified as major barriers for not seeking treatment for RTIs/STIs among the study population. To date, prevention and control of RTIs/STIs, especially among the young married women, is a low priority among rural women. Awareness of women regarding RTIs/STIs certainly helps in prevention and control of those problems. It is a challenging task to raise awareness regarding RTIs/STIs in women because of the social standing of women which distances them from the right source of information and also because of the taboos regarding the discussions on issues like safe sex, unsafe sexual practices, and so forth. The prevention of transmission of infection (primary prevention) is at present receiving increased attention because of the global epidemic of HIV/AIDS and the identification of several sexual infections as risk factors for the spread of HIV [17–19]. Health-seeking behaviour is influenced by a group of factors that can be classified according to cultural and sociodemographic influences, economic conditions, physical and financial accessibility, healthcare services, and the degree of women's autonomy [20, 21].

The role of socioeconomic status in the development of STIs has been highlighted in a number of studies [22–24]. Low socioeconomic status is associated with greater high risk sexual behaviour and this would lead to a higher incidence of STIs [25]. Patients who delayed seeking treatment, including those who treated themselves prior to seeking health care, were female, had friends who had waited before seeking treatment, held misconceptions about the cause of STIs, perceived STIs not to be serious, valued personal autonomy in sexual behavior less, and expected to encounter problems in their relationships if they refused to have sex [26]. Women with a lower educational background delayed seeking care at the first STI provider significantly longer than women with higher education, and urban women sought care significantly earlier than women from rural or remote areas [27].

In India, married women are reluctant to seek medical treatment because of lack of privacy, lack of a female doctor at the health facility, the cost of treatment, and their subordinate social status [28]. This reluctance is exacerbated when symptoms are embarrassing, as they are with RTIs, especially among young women [29]. The health seeking behavior of women is not as improved as desired. The married women are reluctant to seek medical treatment because of lack of privacy, lack of female doctors at the health facility, cost of treatment, and their inferior social status. RTIs have an additional element of shame and humiliation for many women because they are considered unclean. Women do not seek treatment for sexual health problems due to lack of awareness, asymptomatic nature of RTIs, and lack of treatment facilities [30, 31]. Moreover, sexually transmitted infections and reproductive tract infections are the diseases which are associated with some sort of sociocultural stigmas [32].

## 5. Conclusion

Perception of symptoms as normal, lack of female health workers, distance to health facility, and lack of availability of treatment were identified as major barriers for not seeking treatment for RTIs/STIs among the study population. Therefore, more information is required in rural areas through mass media and also more healthcare facilities at the door step of rural women is best-touted option. Likewise, behaviour and communication change and proper sexual health information are the best options to reduce the prevalence of sexual health problems among rural women. There is need for female counselor at each health facility to discuss the sexual health problems and explain correct treatment within a short period of time. Sexual health problems are common and preventable causes of morbidity and serious complications; thus, primary prevention of RTIs/STIs needs to be given high priority. Health educators should adopt this strategy. Integrated approach is strongly suggested for creating knowledge and awareness to control the spread of sexual health problems (including HIV/AIDS) among young people. Appropriate preventive strategies are essential and should be of highest priority because of the potential of such infections to spread particularly among the youth. This raises the necessity to conduct further studies to evaluate the awareness and educate the general population.

## Figures and Tables

**Figure 1:**
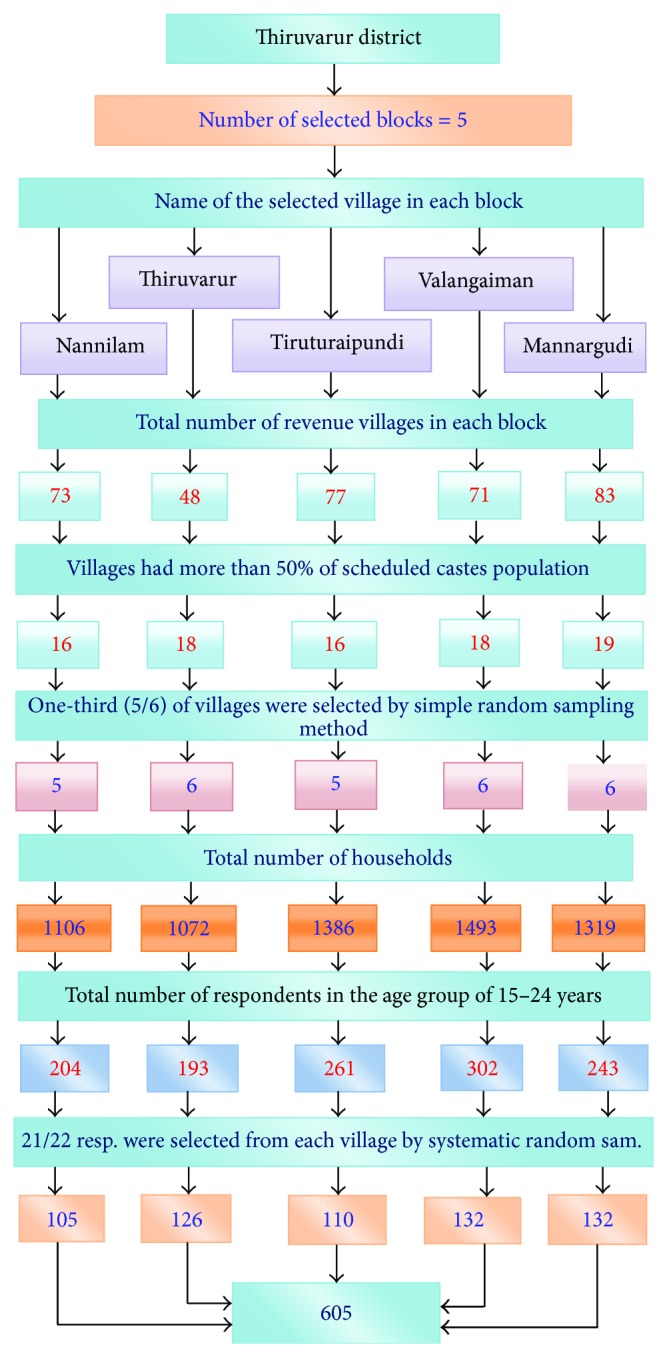


**Table 1:** Percentage of women who experienced/sought treatment of RTIs (multiple responses).

Various types of RTIs	Experienced RTIs		Sought treatment	
	Frequency	Percentage	Frequency	Percentage
Experienced/sought treatment of RTIs	88	14.5	71	80.7
Itching/irritation over vulva	54	8.9	41	75.9
Thick white discharge	28	4.6	27	96.4
Pain in lower abdomen (not related to menses)	28	4.6	17	60.7
Pain during urination	16	2.6	13	81.2
Boils/ulcer around vulva	8	1.3	8	100.0
Swelling in the groin	2	0.3	2	100.0

Total	605		88	

**Table 2:** Percentage distribution of care seeking behaviour of women who experienced RTIs according to their background characteristics.

Background characteristics	Sought treatment		Total	*X* ^2^	*P *
	Frequency	Percentage			
Age of women					
18–20	11	84.6	13	4.29	.117
21–23	42	80.8	52		
24 years	18	78.3	23		
Education of women					
Illiterate	10	76.9	13	9.34	.044
Primary education	18	78.3	23		
Secondary education	38	80.9	47		
Higher secondary+	5	100.0	5		
Occupation of women					
Nonworkers	4	66.7	6	.067	.967
Agricultural laborers	51	81.9	63		
Nonagricultural laborers	16	84.2	19		
Standard of living index					
Low	39	71.9	55	13.64	.008
Medium	31	96.9	32		
High	1	100.0	1		
Age at marriage					
Less than 18 years	3	60.0	5	2.29	.319
18-19 years	41	77.3	53		
20-21 years	23	92.0	25		
22-23 years	4	80.0	5		
Birth order					
First	34	87.2	39	8.32	.015
Second	33	78.6	42		
Third	4	57.1	7		
Exposure to mass media					
More frequently	18	81.8	22	.024	.876
Less frequently	53	80.3	66		
Distance to healthcare facility					
Within one Km	11	91.7	12	2.43	.296
2-3 Km	20	87.0	23		
4 or more Km	40	75.5	53		

Total	71	80.7	88		

**Table 3:** Percentage of women who experienced/sought treatment of STIs (multiple responses).

Various symptoms of STIs	Experienced STIs		Sought treatment	
	Frequency	Percentage	Frequency	Percentage
Women who experienced STIs	53	8.8	41	77.4
Discharge with unpleasant odor	28	4.6	23	82.1
Frequent and uncomfortable urination	22	3.6	18	81.8
Pain during sexual intercourse	19	3.1	14	73.7
Spotting after sexual intercourse	6	1.0	4	66.7

Total	605		53	

**Table 4:** Percentage distribution of care seeking behavior of women who experienced STIs according to their background characteristics.

Background characteristics	Sought treatment		Total	*X* ^2^	*P *
	Frequency	Percentage			
Age of women					
18–20	8	80.0	10	8.78	.077
21–23	27	77.1	35		
24 years	6	75.0	8		
Education of women					
Illiterate	10	66.7	15	11.06	.017
Primary education	7	70.0	10		
Secondary education+	24	85.7	28		
Occupation of women					
Nonworkers	3	75.0	4	1.33	.515
Agricultural laborers	19	63.3	30		
Nonagricultural laborers	19	100.0	19		
Standard of living index					
Low	24	72.7	33	17.17	.006
Medium	8	72.7	11		
High	9	100.0	9		
Age at marriage					
Less than 18 years	1	100.0	1	7.37	.532
18-19 years	22	78.6	28		
20-21 years	13	72.2	18		
22-23 years	5	83.3	6		
Birth order					
First	25	83.3	30	8.95	.029
Second	14	70.0	20		
Third	2	66.7	3		
Exposure to mass media					
More frequently	6	75.0	8	.16	.687
Less frequently	35	77.8	45		
Distance to health care facility					
Within one Km	7	87.5	8	11.23	.027
2-3 Km	10	76.9	13		
4 or more km	24	75	32		

Total	41	77.4	53		

## Abbreviations

HIV: Human immunodeficiency virus
ICDP: International Conference on Population and Development
RH: Reproductive health
RTI: Reproductive tract infection
SC: Scheduled castes
SH: Sexual health
SLI: Standard of living index
SRS: Simple random sampling or systematic random sampling
STI: Sexually transmitted infection
WHO: World Health Organization.
